# Waiting impulsivity in progressive supranuclear palsy-Richardson’s syndrome

**DOI:** 10.3389/fnins.2023.1240709

**Published:** 2023-09-25

**Authors:** Jong Hyeon Ahn, Junmo Kwon, Ji Hye Won, Kyoungseob Byeon, Jinyoung Youn, Hyunjin Park, Jin Whan Cho

**Affiliations:** ^1^Department of Neurology, Samsung Medical Center, Sungkyunkwan University School of Medicine, Seoul, Republic of Korea; ^2^Neuroscience Center, Samsung Medical Center, Seoul, Republic of Korea; ^3^Department of Electrical and Computer Engineering, Sungkyunkwan University, Suwon-si, Republic of Korea; ^4^Center for Neuroscience Imaging Research, Institute for Basic Science, Suwon-si, Republic of Korea; ^5^Department of Computer Engineering, Pukyong National University, Busan, Republic of Korea

**Keywords:** progressive supranuclear palsy, waiting impulsivity, frontal lobe dysfunction, diffusion tensor imaging, nucleus accumbens

## Abstract

**Background:**

Waiting impulsivity in progressive supranuclear palsy-Richardson’s syndrome (PSP-RS) is difficult to assess, and its regulation is known to involve nucleus accumbens (NAc) subregions. We investigated waiting impulsivity using the “jumping the gun” (JTG) sign, which is defined as premature initiation of clapping before the start signal in the three-clap test and compared clinical features of PSP-RS patients with and without the sign and analyzed neural connectivity and microstructural changes in NAc subregions.

**Materials and methods:**

A positive JTG sign was defined as the participant starting to clap before the start sign in the three-clap test. We classified participants into the JTG positive (JTG +) and JTG negative (JTG-) groups and compared their clinical features, microstructural changes, and connectivity between NAc subregions using diffusion tension imaging. The NAc was parcellated into core and shell subregions using data-driven connectivity-based methods.

**Results:**

Seventy-seven patients with PSP-RS were recruited, and the JTG + group had worse frontal lobe battery (FAB) scores, more frequent falls, and more occurrence of the applause sign than the JTG- group. A logistic regression analysis revealed that FAB scores were associated with a positive JTG sign. The mean fiber density between the right NAc core and right medial orbitofrontal gyrus was higher in the JTG + group than the JTG- group.

**Discussion:**

We show that the JTG sign is a surrogate marker of waiting impulsivity in PSP-RS patients. Our findings enrich the current literature by deepening our understanding of waiting impulsivity in PSP patients and introducing a novel method for its evaluation.

## 1. Introduction

Progressive supranuclear palsy (PSP) is a neurodegenerative disease characterized by supranuclear gaze palsy, akinetic rigidity, postural instability, and prominent neuropsychiatric symptoms such as apathy, irritability, and impulsivity (Hoglinger et al., 2017). Impulsivity, defined as the tendency to act prematurely without foresight (Basar et al., 2010), is associated with frequent falls, choking, predicted loss of functional independence, and survival in patients with PSP (Kok et al., 2021). Impulsivity is further classified as stopping and waiting impulsivity. Stopping impulsivity is defined as the inability to cancel an ongoing motor response (Dalley and Robbins, 2017); by contrast, waiting impulsivity is described as a failure to inhibit the initiation of an inappropriate response that is based on incorrect predictions of time or probability (Dalley and Robbins, 2017). Waiting impulsivity can be measured using the 4-choice serial reaction time (4-CSRT) task in humans (Worbe et al., 2014), but there are concerns regarding the suitability of using the 4-CSRT to measure waiting impulsivity in patients with Parkinsonian symptoms who have akinetic rigidity because the task primarily measures motor response time (Seidler et al., 2007; Worbe et al., 2014). In contrast, the three-clap test (TCT) is a simple bedside task commonly used for PSP patients: a patient is asked to clap as fast as possible three times in a row, and the examiner demonstrates the normal three-clap response and gives a start signal before the patient performs the test. Patients with PSP, Parkinson’s disease (PD), or frontal lobe dementia typically show the applause sign (AS) during the TCT (Somme et al., 2013; Schonecker et al., 2019), defined as more than three claps, because they could not stop the behavior, indicating stopping impulsivity (Somme et al., 2013). During the TCT, some PSP patients cannot wait until the start signal, so they jump the gun and start clapping too soon. We denote this behavior as the jumping the gun (JTG) sign and speculate that it might represent waiting impulsivity.

In addition to having clinically different manifestations, stopping and waiting impulsivity are associated with distinct neural network systems. Stopping impulsivity is dependent on dorsostriatal mechanisms (Dalley and Robbins, 2017), whereas waiting impulsivity is mainly regulated by the nucleus accumbens (NAc) and its connected neural networks (Dalley and Robbins, 2017). The corticostriatal pathway, which includes the ventral striatum (specifically the nucleus accumbens, or NAc) and several regions of the prefrontal cortex (medial orbitofrontal, lateral orbitofrontal, lateral, ventromedial, and ventrolateral), as well as the cingulate gyrus, plays a pivotal role in managing waiting impulsivity (Dalley and Robbins, 2017). Research on the NAc in PSP patients found lower choline acetyltransferase levels (Warren et al., 2005), decreased volume, and reduced nodal degree compared with healthy controls (Abos et al., 2019), indicating that both functional and structural changes in the NAc occur in PSP patients. The NAc is known to be divided into two distinct regions, the core and the shell, which have opposing roles in the regulation of waiting impulsivity. It has been observed that excitotoxic lesions (excessive exposure to the neurotransmitter glutamate or overstimulation of its membrane receptors) in the NAc core lead to increasing waiting impulsivity (Cardinal et al., 2001), and the shell regions antagonize that increase (Sesia et al., 2008; Dalley and Robbins, 2017). Despite their opposite roles in the regulation of waiting impulsivity, previous studies on PSP did not separately analyze the NAc core and shell (Warren et al., 2005; Biundo et al., 2015; Ikuta et al., 2018; Abos et al., 2019). A given brain region can be parcellated into smaller subregions using various imaging features in a data-driven fashion (Xia et al., 2017; Zhao et al., 2018). Such a subdivision procedure is unbiased because it does not involve manual specification of the subregions. A recent study by Zhao et al. (2018) successfully parcellated the NAc into core and shell regions using connectivity-based methods. Building on that previous research, we investigated microstructural changes and changes in connectivity between the core and shell of the NAc and their associated structures in PSP patients using diffusion tensor imaging (DTI), which is a sensitive imaging modality (Wang et al., 2010; Spotorno et al., 2019).

In this study, we had three objectives: to investigate the presence of waiting impulsivity using the JTG sign; to compare the clinical features of PSP-Richardson’s syndrome (PSP-RS) patients with and without the JTG sign; and to identify the neural connectivity and microstructural changes associated with waiting impulsivity in PSP-RS patients.

## 2. Materials and methods

### 2.1. Participants and clinical assessments

**FIGURE 1:**
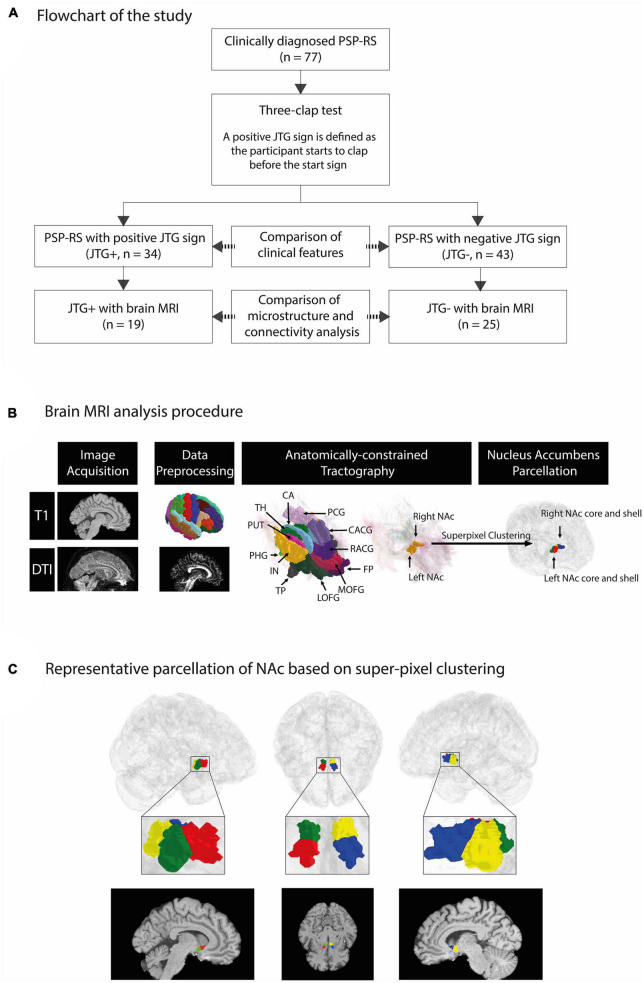
An overview of our framework to segment the NAc into core/shell region and representative parcellation of NAc based on super-pixel clustering. A total of 77 patients with PSP-RS were enrolled in the study. We compared the demographic and clinical characteristics of the participants based on the presence of the JTG sign, with 34 classified as JTG + and 43 as JTG-. Out of the 77 participants, 44 underwent T1-weighted and DWI MRI and were included in the brain MRI analysis, with 19 classified as JTG + and 25 as JTG- **(A)**. FreeSurfer was applied to T1-weighted MRI data to parcellate regions based on the Desikan–Killiany atlas. We used the Mrtrix3 framework to preprocess diffusion-weighted MRI data and performed anatomically constrained tractography to compute streamline density between the NAc and 14 target regions. Finally, super-pixel clustering was applied based on the streamline density with each target region to identify the core and shell regions of the left NAc and right NAc **(B)**. In the left column of panel **(C)**, red and green denote the core and shell subregions, respectively, of the left NAc. In the right column, blue and yellow denote the core and shell subregions, respectively, of the right NAc. The middle column shows the subregions of both the left and right NAc in an axial view **(C)**. PSP-RS, progressive supranuclear palsy-Richardson’s syndrome; JTG, jumping the gun; AMY, amygdala; CA, caudate; CACG, caudal anterior cingulate gyrus; RACG, rostral anterior cingulate gyrus; PCG, posterior cingulate gyrus; PUT, putamen; TH, thalamus; LOFG, lateral orbitofrontal gyrus; MOFG, medial orbitofrontal gyrus; FP, frontal pole; HIP, hippocampus; IN, insula; PHG, parahippocampal gyrus; TP, temporal pole; NAc, nucleus accumbens.

We recruited patients who were diagnosed with probable PSP-RS based on the Movement Disorder Society criteria for clinical diagnosis of PSP (Hoglinger et al., 2017). The TCT was applied to detect the presence of the JTG sign. The examiner demonstrated TCT and asked the patients to clap three times as quickly as possible, following a given start signal from the examiner (Supplementary Video 1). Each patient underwent the test once, administered by one of the three movement disorder specialists (JA, JY, and JC). A consensus on the positive sign was reached prior to the study. The performance of the participant was classified as normal when they started after the start signal and clapped only three times. A positive JTG sign was recorded when a participant started to clap before the start signal. Positive AS was recorded when a participant clapped more than three times. The age, sex, disease duration, PSP-rating scale (PSPRS) (Golbe and Ohman-Strickland, 2007), and frontal assessment battery (FAB) (Dubois et al., 2000) of each participant were investigated. The medications that patients were taking at the time of the investigation were thoroughly examined. These medications included levodopa, dopamine agonists, antidepressants, antipsychotics, and acetylcholinesterase inhibitors. Additionally, we calculated the Levodopa Equivalent Daily Dose (LEDD) for each patient (Schade et al., 2020). The flowchart for participant selection is given in Figure 1A. This study was approved by the Institutional Review Board of Samsung Medical Center (IRB No. SMC-2022-11-138), Seoul, Republic of Korea, and written informed consent was obtained from all participating patients.

### 2.2. MRI data acquisition and data preprocessing

Brain MRI scans were conducted simultaneously with their clinical evaluations (4.0 ± 2.4 days). T1-weighted MRI and diffusion-weighted imaging (DWI) MRI data were acquired at Samsung Medical Center using a Philips Achieva 3.0 Tesla MRI scanner. The T1-weighted structural MRI scans were collected using an accelerated turbo field echo sequence (SENSE with the acceleration factor of 1) with the following imaging parameters: repetition time (TR) = 9.9 ms; echo time (TE) = 4.6 ms; flip angle = 8°; field-of-view (FOV) = 240 mm× 240 mm; voxel resolution = 0.5 mm× 0.5 mm× 0.5 mm; matrix size = 480 × 480; and number of slices ranging from 310 to 365. DWI scans were obtained using a single-shot echo-planar imaging sequence with these imaging parameters: TR = 9,800 ms; TE = 87 ms; flip angle = 90°; FOV = 220 mm× 220 mm; voxel resolution = 1.719 × 1.719 × 2.0 mm^3^; matrix size = 128 × 128; number of slices = 70; number of gradient orients = 45; single-shell *b*-value = 600 s/mm^2^. T1-weighted and DWI MRI data were processed using Mrtrix3 (Tournier et al., 2019). The 5ttgen command in Mrtrix3 was used to process T1-weighted data, and it included the preprocessing steps found in the *recon-all* command in FreeSurfer (Fischl, 2012). DWI data preprocessing involved denoising, Gibbs ringing artifact correction, eddy current correction, motion correction, and N4 bias field correction. DTI was estimated from the preprocessed DWI data using Mrtrix3 (Tournier et al., 2019). The fractional anisotropy (FA) and mean diffusivity (MD) values were derived from the DTI and used to compare microstructural differences between the core and shell subregions of the NAc.

### 2.3. Tractography and structural connectivity

Structural connectivity was measured in terms of streamlines in the whole-brain voxel-to-voxel tractography using preprocessed DWI data in Mrtrix3 (Tournier et al., 2019). First, we estimated the response function of gray matter, white matter, and cerebrospinal fluid. Then, the preprocessed DWI data were resampled onto a 1-mm^3^ isotropic voxel grid space to ensure easier co-registration with high-resolution T1 data and fine-grained computation of streamlines (Avants et al., 2011). The fiber orientation density (FOD) was calculated using single-shell multi-tissue-constrained spherical deconvolution (Tournier et al., 2019). Additionally, we corrected tissue-specific bias fields and intensity inhomogeneity of the FOD (Raffelt et al., 2017). We used the anatomical-constrained tractography method (Smith et al., 2012) with the T1 data-derived segmentation results for cortical gray matter, sub-cortical gray matter, white matter, cerebrospinal fluid, and pathological tissue. The segmentation maps were registered onto the b0 image of the DWI data. To perform tractography, we used iFOD2 probabilistic tractography with 10 million seeds placed at the boundary between gray matter and white matter, a maximum streamline length of 250 mm, and a FOD amplitude cutoff of 0.06 (Tournier et al., 2010). The obtained streamlines were then refined using the Sift2 algorithm constrained by five-tissue segmentation (Smith et al., 2015).

### 2.4. Nucleus accumbens parcellation

The NAc region was divided into tentative core and shell subregions based on prior studies (Xia et al., 2017; Zhao et al., 2018). A data-driven super-pixel clustering algorithm using a zero parameter version of simple linear iterative clustering (Achanta et al., 2012) was applied to voxels in the NAc, as defined in the DWI space (i.e., 1-mm^3^ isotropic resolution), using streamline features. The Desikan–Killiany atlas was used to specify the NAc and the following 14 target regions: amygdala, caudate, caudal anterior cingulate gyrus, rostral anterior cingulate gyrus, posterior cingulate gyrus, putamen, thalamus, lateral orbitofrontal gyrus, medial orbitofrontal gyrus (MOFG), frontal pole, hippocampus, insula, parahippocampal gyrus, and temporal pole (Zhao et al., 2018). The 14 target regions were chosen based on a previous study (Zhao et al., 2018). Streamlines between each voxel in the NAc and the 14 target regions were computed using the track density imaging method, resulting in a 14-element vector that was used to cluster all the voxels in the NAc into two regions in an unsupervised manner. The superior subregion was noted as the core, and the inferior subregion was noted as the shell. The MRI analysis procedure is outlined in Figure 1B.

### 2.5. Statistical analyses

All continuous variables are presented in mean ± standard deviation format. Data were examined for normality using the Kolmogorov–Smirnov test. We divided the participants according to the presence of the JTG sign (JTG + and JTG-). The group comparison of baseline data was performed using the chi-square test or student’s *t*-test, depending on the variable type. To determine the factors associated with the JTG sign, multivariate logistic regression analyses were conducted using covariates with a *p*-value of ≤ 0.2 in the univariate analyses. We computed the mean fiber density from the NAc subregion (core or shell) to the 14 target regions as the sum of the streamlines seeded from all the voxels in the subregion to the target region divided by the total number of voxels in the subregion. Multiple comparisons were corrected using the Benjamin-Hochberg false discovery rate (FDR) correction. All statistical analyses were performed in IBM SPSS (version 28.0; IBM Inc., Armonk, NY, USA) software for Windows.

## 3. Results

### 3.1. Participants

We included 77 PSP-RS patients in this study, and their demographics and clinical characteristics are presented in Table 1. Among the participants, 34 (44.2%) patients with PSP showed the JTG sign, and 43 (56.8%) did not. The mean age, sex, disease duration, and years of education were comparable in the two groups. The PSPRS total and sub-scores did not differ between the JTG + and the JTG- groups. The JTG + group had a lower FAB total (5.5 ± 3.8 vs. 9.9 ± 4.2, *p* < 0.001), across all subscores, except for the conflicting instructions. They also had a higher fall frequency (2.4 ± 1.3 vs. 1.8 ± 1.5, *p* = 0.045), as measured by PSPRS Q5 (Falls). Additionally, the JTG + group had a higher proportion of patients who showed the AS (73.5% vs. 37.2%, *p* = 0.002). Regarding medication, we found no significant differences between the two groups. In the logistic regression analysis, only the FAB score was associated with the JTG sign (Table 2).

**TABLE 1:** Demographic and clinical characteristics of patients with progressive supranuclear palsy - Richardson’s syndrome.

	JTG+ (*n* = 34)	JTG− (*n* = 43)	*P*-value
Age, years	71.6 ± 6.4	72.1 ± 8.3	0.792
Sex, male (%)	15 (44.1)	13 (30.2)	0.208
Disease duration, m	46.6 ± 28.1	67.8 ± 8.4	0.993
Education, years	10.3 ± 4.5	10.6 ± 5.0	0.423
FAB	5.5 ± 3.8	9.9 ± 4.2	<**0**.**001**
Similarities	0.8 ± 0.8	1.3 ± 1.0	**0**.**018**
Lexical fluency	0.8 ± 0.8	1.6 ± 1.8	**0**.**007**
Luria’s test	0.5 ± 0.7	1.4 ± 1.2	<**0**.**001**
Conflicting instructions	0.8 ± 1.0	1.2 ± 1.1	0.059
Go-No go	0.7 ± 1.0	1.8 ± 1.3	<**0**.**001**
Grasp reflex	1.9 ± 1.0	2.5 ± 0.7	**0**.**010**
**PSPRS**			
History	8.2 ± 3.2	7.4 ± 4.6	0.199
Mentation	2.8 ± 3.2	1.9 ± 3.1	0.107
Bulbar	2.7 ± 1.4	2.6 ± 1.6	0.419
Ocular	5.4 ± 3.0	5.3 ± 3.6	0.456
Limb	3.9 ± 1.5	3.7 ± 2.3	0.386
Gait	10.9 ± 3.9	9.4 ± 5.0	0.071
Total	33.7 ± 11.5	30.3 ± 17.0	0.160
Fall frequency ^a^	2.4 ± 1.3	1.8 ± 1.5	**0**.**045**
AS, *n* (%)	25 (73.5)	16 (37.2)	**0**.**002**
Levodopa, *n* (%)	34 (100.0)	43 (100.0)	>0.999
Dopamine agonists, *n* (%)	1 (2.9)	2 (4.7)	>0.999
Antidepressants	6 (17.6)	10 (23.3)	0.547
Antipsychotics	2 (5.9	3 (7.0)	0.847
AChEI	11 (25.6)	15 (34.9)	0.515
LEDD (mg/day)	348.5 ± 145.4	364.0 ± 162.0	0.666

JTG, jumping the gun sign; PSPRS, Progressive Supranuclear Palsy Rating Scale; FAB, frontal assessment battery; AS, applause sign; AchEi, acetylcholine esterase inhibitors; LEDD, levodopa equivalent daily dose.

^a^Based on PSPRS Q5 (Falls): 0, None in the past year; 1, <1 per month; 2, 1–4 per month; 3, 5–30 per month; 4, >30 per month.

Data are presented as the mean ± standard deviation.

Bold values denote statistical significance (*p* < 0.05).

**TABLE 2:** Logistic regression analysis for predicting the jumping the gun sign in PSP-RS patients.

	B	Exp (B)	95% CI for B		*P*-value
			Lower	Upper	
Age	0.000	1.000	0.924	1.083	0.995
Disease duration	-0.008	0.992	0.973	1.011	0.410
Sex (male)	0.211	1.235	0.402	3.797	0.713
FAB	-0.281	0.755	0.646	0.882	<**0**.**001**
AS	0.045	1.046	0.862	1.271	0.647
PSPRS history	-0.232	0.793	0.609	1.032	0.084
PSPRS mentation	0.180	1.197	0.898	1.595	0.220
PSPRS gait	0.042	1.043	0.888	1.224	0.609
Fall frequency ^a^	0.411	1.508	0.869	2.617	0.144

FAB, frontal assessment battery; AS, applause sign; PSPRS, Progressive Supranuclear Palsy Rating Scale.

^a^Based on PSPRS Q5 (Falls): 0, None in the past year;1, <1 per month; 2, 1–4 per month; 3, 5–30 per month; 4, >30 per month.

Bold values denote statistical significance (*p* < 0.05).

### 3.2. The NAc subregion analysis

Among the 77 participants, 44 underwent T1-weighted and DWI MRI and were subsequently included in the brain MRI analysis. The patients included in the brain MRI analysis had longer disease durations and higher mentation subscores on the PSPRS, while no other significant differences were observed (Table 1). Nineteen of them were positive for the JTG sign, and 25 did not have the JTG sign. Consistent with the findings in the overall study population, no significant differences were observed between the two groups in age, sex, disease duration, years of education, or PSPRS total scores and sub-scores. The JTG-positive group showed a lower FAB score, a higher score in the fall frequency question, and a higher prevalence of positive AS, reflecting the patterns observed in the overall study population (Table 2).

**FIGURE 2:**
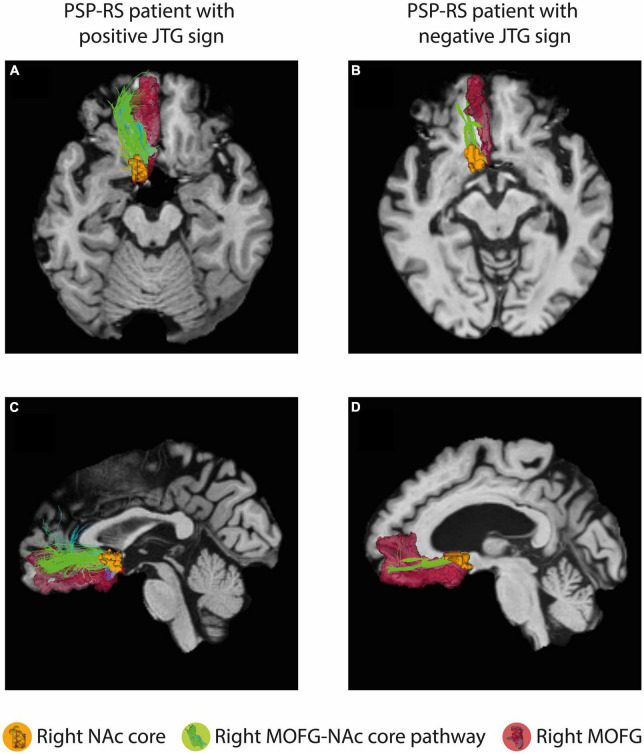
The connectivity analysis showed a significant difference in the mean fiber density between the right NAc core and right MOFG in the JTG + group compared with that in the JTG- group (*p* = 0.001, *p*_FDR = 0.017). **(A,C)** Represent axial and sagittal images of the JTG+ group, showing the connectivity between the right NAc core and the right MOFG, as well as the right MOFG-NAc core pathway. **(B,D)** Display the corresponding images for the JTG- group. NAc, nucleus accumbens; MOFG, medial orbitofrontal gyrus; JTG, jumping the gun; FDR, false discovery rate.

Figure 1C presents a representative NAc core and shell from the JTG- group, a male subject aged 63 years with a disease duration of 99 months. The volumes of the NAc core and shell did not differ significantly between the JTG + and JTG- groups (Table 3). The mean FA value of the right NAc core in the JTG + group was significantly higher than that of the JTG- group (0.205 ± 0.051 vs. 0.174 ± 0.040, uncorrected *p* = 0.038). However, after adjusting for the FDR, the significance disappeared (*p*_FDR = 0.153). Otherwise, no significant differences in the FA and MD values were observed between the JTG + and JTG- groups (Table 3). We compared the mean fiber density between the NAc core/shell and the 14 brain regions and found that the mean fiber density connecting the right NAc core and right MOFG in the JTG + group was significantly higher than that in the JTG- group (*p* = 0.001, *p*_FDR = 0.017) (Figure 2). None of the other comparisons yielded any significant differences (Supplementary Figure 1).

**TABLE 3:** Comparison of the volumes, mean FA, and mean MD values of the NAc core and NAc Shell in the JTG + and JTG- groups.

Metric	Cluster	JTG+ group (*n* = 19)	JTG− group (*n* = 25)	Uncorrected *P*-value	FDR-adjusted *P*-value
Volume in mm^3^	Left NAc core	131.316 ± 24.885	132.320 ± 41.296	0.927	0.927
	Left NAc shell	195.526 ± 31.991	186.240 ± 47.986	0.480	0.640
	Right NAc core	197.053 ± 61.078	174.880 ± 37.636	0.155	0.620
	Right NAc shell	238.000 ± 54.264	254.720 ± 59.882	0.356	0.640
Mean FA	Left NAc core	0.191 ± 0.033	0.167 ± 0.054	0.077	0.154
	Left NAc shell	0.188 ± 0.031	0.199 ± 0.086	0.554	0.554
	Right NAc core	0.205 ± 0.051	0.174 ± 0.040	**0**.**038**	0.153
	Right NAc shell	0.200 ± 0.037	0.188 ± 0.052	0.387	0.516
Mean MD	Left NAc core	0.001170 ± 0.000276	0.001415 ± 0.000586	0.080	0.320
	Left NAc shell	0.001416 ± 0.000330	0.001488 ± 0.000517	0.590	0.590
	Right NAc core	0.001324 ± 0.000365	0.001245 ± 0.000291	0.454	0.590
	Right NAc shell	0.001335 ± 0.000378	0.001470 ± 0.000365	0.252	0.504

Volumes are represented in mm^3^.

Values are reported as the mean ± standard deviation.

*P*-values were obtained using two-sample *T*-tests and corrected with the Benjamin-Hochberg false discovery rate.

JTG, jumping the gun sign; NAc, nucleus accumbens; FA, fractional anisotropy; MD, mean diffusivity.

Bold values denote statistical significance (*p* < 0.05).

## 4. Discussion

In this study, we have introduced the possibility that the JTG sign is an indicator of waiting impulsivity in patients with PSP-RS. Our results show that 44.2% of the patients with PSP-RS exhibited waiting impulsivity as assessed by the JTG sign, and having the JTG sign was associated with frequent falls and frontal lobe dysfunction, despite comparable demographic features and motor symptoms. Furthermore, we observed a relationship between JTG sign positivity and connectivity between the right NAc core and right MOFG. This suggests that the JTG sign could serve as a valuable indicator of waiting impulsivity in patients with PSP-RS.

Impulsivity is a common non-motor symptom in patients with PSP and can present in various forms, such as impulsive decision-making, impulsive eating, compulsive behavior, and reckless gait (Burrell et al., 2014). In this study, we used the TCT, which is a popular bedside neurological test for patients with PSP. Conventionally, impaired performance on the TCT is defined using the AS, which is characterized as being unable to stop clapping and is thought to indicate stopping impulsivity, reflecting an impaired stop process generated by the inferior frontal cortex and activation of the subthalamic nucleus (Schonecker et al., 2019). Unlike previous studies, we focused on waiting impulsivity and successfully demonstrated that the JTG sign, also derived from the TCT, is a useful tool for assessing waiting impulsivity in PSP-RS patients. Furthermore, we found that 44.2% of our PSP-RS patients exhibited waiting impulsivity, though direct comparison with previous studies is challenging because we focused solely on waiting impulsivity, whereas previous studies did not measure waiting impulsivity separately.

In this study, we hypothesized that the presence of the JTG sign would serve as an indicator of waiting impulsivity, with the NAc playing a pivotal role in its regulation in patients with PSP-RS. We evaluated our hypothesis using brain MRI analyses, and they supported our proposition. The mean FA value of the right NAc core was found to be significantly higher in the JTG + group compared to the JTG- group. This suggests a potential preservation of the microstructure within the right NAc core in the JTG + group. However, this significance disappeared after the correction for multiple comparisons was applied. This underscores the need for additional studies to thoroughly examine the association between microstructural changes in the NAc and waiting impulsivity. Addition, we discovered that the JTG + group displayed increased connectivity between the NAc core and the right MOFG. This finding suggests that the NAc core plays a significant role in regulating waiting impulsivity in PSP-RS patients. Our results are largely consistent with those of previous studies. For instance, Biundo et al. (2015) showed that increased FA in the accumbofrontal tract had a significant positive correlation with impulsivity, suggesting that accumbofrontal track integrity contributes to impulsivity in PD (Ikuta et al., 2018). Wang et al. (2019) reported that the medial OFG-NAc core pathway is more important than the lateral OFG-NAc core pathway in decisional impulsivity. Zanigni et al. (2016) reported that the NAc was more significantly involved in PSP than in PD, further supporting the notion that the NAc plays an important role in PSP. Consistent with previous research, our results show that the JTG + group had higher FA in the right NAc core and greater mean fiber connectivity between the right NAc core and right MOFG than the JTG- group. The different roles of the right and left NAc in impulsivity regulation remain unclear. Studies of PD patients found that a reduction in the right NAc volume was associated with impulsive control disorders and response disinhibition (O’Callaghan et al., 2013; Biundo et al., 2015). Conclusive results are limited because previous studies did not separately analyze the NAc core and shell. One of the strengths of our study is our separate analysis of the NAc core and shell, which is unique among studies and allows us to investigate the opposing roles these subregions play in regulating waiting impulsivity.

The results of our univariable and logistic regression analyses indicate that the JTG + group exhibited more severe frontal lobe dysfunction than the JTG- group. In the JTG + group, the FAB subscores for conceptualization, mental flexibility, motor programming, inhibitory control and environmental autonomy was significantly lower, and the coexistence of AS was significantly higher compared to the JTG- group. These findings suggest that both the NAc and frontal dysfunction play roles in the regulation of waiting impulsivity (Dalley et al., 2011; Wang et al., 2019). Cognitive decline, specifically frontal lobe dementia, is a clinical characteristic of PSP-RS that affects 40–62% of patients with PSP (Brown et al., 2010), and such patients can also exhibit additional frontal lobe release signs, including paratonia, motor preservations, grasping, and utilization behavior (Kojovic and Bhatia, 2019). Consequently, when patients test positive for the JTG sign, clinicians should assess their frontal lobe dysfunction and provide timely and appropriate management. Our results did not focus on structural changes in the frontal lobe, so further research is necessary to better understand the top-down regulation and role of the frontal lobe in the regulation of waiting impulsivity in PSP patients.

Frequent falls are a core clinical feature of PSP-RS, and multiple factors contribute to the occurrence of falls, including parkinsonism, freezing of gait, and reduced postural reflexes (Brown et al., 2020; Bluett et al., 2021). In addition, psychiatric symptoms, particularly impulsivity, highly contribute to the frequency of falls in people with PSP (Smulders et al., 2014; Brown et al., 2020; Mateen et al., 2021), and that was also confirmed in this study. The JTG + group was more likely to fall than the JTG- group, despite similar disease duration, PSPRS scores, and gait subscores. Thus, waiting impulsivity (as measured by the JTG sign) is a clinically significant factor associated with falls in PSP-RS. PSP patients with waiting impulsivity tend to attempt walking or standing before the arrival of an assistant, even if the assistant is only a few seconds away, which can increase the risk of falls (Burrell et al., 2014). Therefore, if PSP patients exhibit the JTG sign, clinicians should recognize the increased risk of falls and take appropriate preventive measures.

This study has several limitations. First, not all the participants underwent brain MRI. Those who were included in the MRI analysis had a longer disease duration compared to those who were excluded. However, when the patients were split into two groups based on the presence of the JTG sign, both among those who underwent MRI and those who did not, the characteristics showing differences were consistent. Second, there is no gold standard for checking waiting impulsivity or motor impulsivity in Parkinsonian patients, especially PSP patients with akinetic rigidity, bradykinesia, and cognitive decline. Several tests and scales have been suggested, but they have not been validated. Therefore, we investigated only the JTG sign and brain MRI to probe its association with changes in the NAc core, which is the most important structure known to regulate impulsivity. Third, our study did not include healthy controls, making it difficult to draw comparisons with the normal population. However, several studies of PD in humans have consistently shown that the NAc, particularly the core, plays a critical role in regulating waiting impulsivity. Despite the lack of a healthy control group in this study, these prior findings lend support to the notion that the NAc is indeed an important structure in the regulation of waiting impulsivity. Fourth, this study included only patients with PSP-RS, and therefore our findings might not be applicable to patients with other types of PSP. Moreover, the JTG is not a specific sign for PSP, it can also manifest in other neurodegenerative diseases characterized by frontal lobe dysfunction including frontal lobe dementia, corticobasal syndrome, and Parkinson’s disease dementia like the AS (Schonecker et al., 2019). Further research is needed to investigate the broader implications of the JTG sign and waiting impulsivity in PSP.

## 5. Conclusion

In this study, we identified the JTG sign as a surrogate marker of waiting impulsivity in PSP-RS patients. Patients with the JTG sign had poorer frontal lobe function and more frequent falls than those without the JTG sign. Furthermore, the results show that a positive JTG sign is associated with microstructural changes, neural connectivity, frontal lobe dysfunction, and frequent falls. Our findings enrich the current literature by deepening our understanding of waiting impulsivity in PSP patients and introducing a novel method for evaluating it.

## Data availability statement

The raw data supporting the conclusions of this article will be made available by the authors, without undue reservation.

## Ethics statement

This research was reviewed and approved by the Institutional Review Board of Samsung Medical Centre (IRB No. SMC-2022-11-138). The studies were conducted in accordance with the local legislation and institutional requirements. The participants provided their written informed consent to participate in this study.

## Author contributions

JA, JK, HP, and JC: conceptualization, methodology, and project administration. JA, JK, and HP: formal analysis. HP and JC: supervision. JA and JK: writing—original draft. All authors contributed to data curation, investigation, and writing—review and editing and approved the submitted version.
