# Supplementary figures and images for: Waiting impulsivity in progressive supranuclear palsy-Richardson’s syndrome
Source: Front Neurosci. 2023 Sep 25;17:1240709. doi: 10.3389/fnins.2023.1240709 (PMC10560850; doi:10.3389/fnins.2023.1240709)

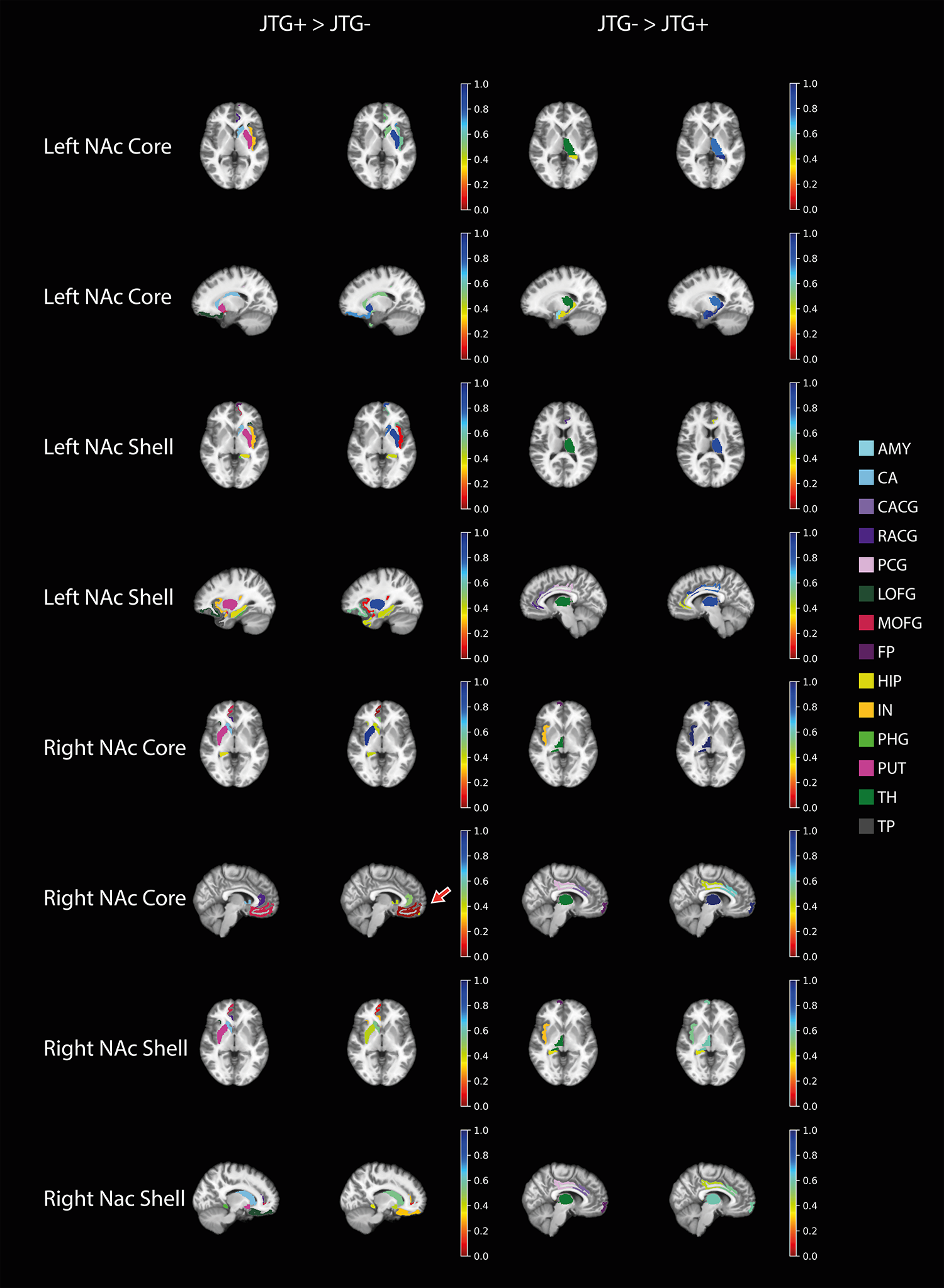

Supplement: Supplementary file 2 [file Image_1.TIFF]
